# Reply to: ‘Letter to the Editor: Neuropathies related to hepatitis E virus infection: First reported case in Europe’

**DOI:** 10.1111/ene.16137

**Published:** 2023-11-09

**Authors:** Paolo Ripellino, Claudio Gobbi

**Affiliations:** ^1^ Department of Neurology Neurocenter of Southern Switzerland EOC Lugano Switzerland; ^2^ Faculty of Biomedical Sciences Università della Svizzera Italiana Lugano Switzerland

Dear Editor,

We thank Bravo Urbieta et al. for their comments.

We fully agree that absence of evidence does not equate to evidence of absence of a relationship between hepatitis E virus (HEV) infection and Bell's palsy, and further studies should explore this association in different geographical areas and settings. However, in the hierarchy of the evidence‐based medicine pyramid, results obtained in two different matched‐control studies [1, 2] are needed to confirm observations emerging from isolated case reports [3]. As the Spanish may be different from the Swiss situation with regards to HEV strain genotypes, we encourage Bravo Urbieta et al. to set up a case–control study with adequate statistical power to finally prove or rebut the association between HEV infection and Bell's palsy because we agree that our study was not powered to detect such association.

Bravo Urbieta et al. conclude their letter suggesting ‘the inclusion of serological screening for hepatitis E in the initial evaluation of patients with peripheral facial paralysis, as recommended by clinical practice guidelines’. We point out, however, that the European Association for the Study of the Liver (EASL) guidelines [4] ***do not*** recommend to test for HEV in Bell's palsy. EASL guidelines ‘***recommend*** HEV testing, irrespective of liver function test results, in patients presenting with Neuralgic Amyotrophy (level of evidence: B1) and Guillain‐Barré Syndrome (level of evidence: B1)’ and ‘***suggest*** HEV testing for patients with encephalitis/myelitis (level of evidence: C2)’. In the 2018 edition of the EASL guidelines, Bell's palsy is not included in the list of conditions potentially requiring HEV testing because of lack of studies (table 5 of reference 8). We also refer to the German/Austrian/Swiss [5] neurological guidelines, in which there is no mention of HEV as a potential trigger of Bell's palsy because of lack of evidence.

We should also like to comment on the potential role for ribavirin in the treatment of Bell's palsy. Interestingly, in the case report by Bravo Urbieta et al. [3], the HEV+ patient was treated with steroids and acyclovir, but not ribavirin, and he had a good outcome (‘asymptomatic without sequelae’) already after 1 month. This raises the question of whether using ribavirin for this disease in immunocompetent patients is of any clinical value, considering that ribavirin treatment can cause side effects (mainly anaemia) and at the same time increases medical costs. In fact, according to the EASL guidelines, ‘acute HEV infection does not usually require antiviral therapy (class of evidence: A) and ribavirin treatment may be considered in cases of severe acute hepatitis E or acute‐on‐chronic liver failure (class of evidence: C2)’.

Thus, we are not changing our conclusions and, consequently, we do not recommend clinical routine testing for HEV. Testing of HEV, however, may be helpful in patients with Bell's palsy and unexplained increased liver enzyme levels, especially if they are immunosuppressed (as for instance solid‐organ transplant recipients), because these patients could theoretically profit from ribavirin to increase viral clearance.

Finally, we encourage other neurologists and hepatologists to study neuropathies also evaluating potential relationships with HEV.

## AUTHOR CONTRIBUTIONS

**Paolo Ripellino:** Conceptualization; data curation; formal analysis; investigation; methodology; resources; supervision; writing – original draft; writing – review and editing. **Claudio Gobbi:** Conceptualization; investigation; writing – original draft; methodology; writing – review and editing; formal analysis; data curation; supervision; resources.

## CONFLICT OF INTEREST STATEMENT

There are no competing interests for the authors.

## Data Availability

No data were collected from human or animal subjects (or participants) in this study. The data that support the findings of this study are available at the websites given in references cited in the letter. These data are available in the public domain.

## References

[1] Fritz‐Weltin M , Niedermeier L , Frommherz E , et al. Hepatitis E virus and Bell's palsy. Eur J Neurol. 2022;29:820‐825. 34748257 10.1111/ene.15175

[2] Ripellino P , Lascano AM , Scheidegger O , et al. Neuropathies related to hepatitis E virus infection: a prospective, matched case‐control study. Eur J Neurol. 2023;1‐7. doi:10.1111/ene.16030 PMC11235744 37548584

[3] Bravo Urbieta J , Martin Cascon M , Aleman BS . Letter to the editor in response to ‘Hepatitis E virus and Bell's palsy. Eur J Neurol. 2023;30:1551‐1552. 36751988 10.1111/ene.15724

[4] European Association for the Study of the Liver . Electronic address eee, European Association for the Study of the L. EASL clinical practice guidelines on hepatitis E virus infection. J Hepatol. 2018;68:1256‐1271. 29609832 10.1016/j.jhep.2018.03.005

[5] Heckmann JG , Lang C , Glocker FX , et al. Therapie der idiopathischen Fazialisparese (Bell's palsy), S2k‐Leitlinie. 2022. Deutsche Gesellschaft für Neurologie (Hrsg), *Leitlinien für Diagnostik und Therapie in der Neurologie Online: wwwdgnorg/leitlinien*.

